# Commentary: Blood flow restriction combined with resistance training on muscle strength and thickness improvement in young adults: a systematic review, meta-analysis, and meta-regression

**DOI:** 10.3389/fphys.2024.1486727

**Published:** 2024-10-17

**Authors:** Victor S. de Queiros, Rodrigo R. Aniceto, Nicholas Rolnick, Magno F. Formiga, João G. Vieira, Breno Guilherme de Araújo Tinôco Cabral, Paulo Moreira Silva Dantas

**Affiliations:** ^1^ Graduate Program in Healthy Science, Federal University of Rio Grande do Norte, Natal-RN, Brazil; ^2^ Study and Research Group in Biomechanics and Psychophysiology of Exercise, Federal Institute of Education, Science and Technology of Rio Grande do Norte, Nova Cruz-RN, Brazil; ^3^ Graduate Program in Cognitive Neuroscience and Behavior, Federal University of Paraiba, João Pessoa, Brazil; ^4^ Department of Exercise Science and Recreation, CUNY Lehman College, Bronx, NY, United States; ^5^ The Human Performance Mechanic, Bronx, NY, United States; ^6^ Graduate Program in Physiotherapy and Functioning, Department of Physiotherapy, Federal University of Ceará, Fortaleza, Brazil; ^7^ Graduate Program in Physical Education, Federal University of Juiz de Fora, Juiz de Fora, Brazil; ^8^ Graduate Program in Physical Education, Federal University of Rio Grande do Norte, Natal-RN, Brazil

**Keywords:** blood flow restriction training, KAATSU, vascular occlusion, strength training, muscle hypertrophy

## Introduction

Systematic reviews (SRs) are studies that aim to provide a comprehensive and impartial synthesis of multiple studies on a given topic, bringing together “all” relevant evidence in a single document to answer specific research questions (Rother, 2007; Aromataris and Pearson, 2014). SRs are widely useful for health professionals who has limited time to read several articles on a given topic, but carry out their practice based on evidence. Therefore, it is essential that SRs are conducted with the methodological rigor expected of any research. Recently, a group of researchers conducted a SR and meta-analysis that aimed to evaluate the effects of resistance training (RT) with blood flow restriction (BFR) on strength and “muscle thickness” in healthy individuals (Ma et al., 2024). The topic explored in this study is highly relevant and valuable, and we commend the authors for their efforts. However, we believe that additional detail and attention to certain methodological aspects could enhance the interpretation of the results. In this document, we will be discussing some points that may have contributed to erroneous conclusions about the results presented in the study.

### Study selection

It is recommended that eligibility criteria for study selection be based on the PICOS elements defined by the review question (Aromataris and Pearson, 2014). Although the researchers sought to follow this approach, crucial aspects were not adequately reported. We noted that some details regarding the interventions, such as load used during BFR training, duration, frequency, and characteristics of comparator conditions, were not fully reported. This omission makes it difficult to understand the criteria for study selection.

Assuming that the authors did not apply restrictions regarding the intervention time, it is possible to identify that certain studies (Yasuda et al., 2010; Fujita et al., 2008; Abe et al., 2005) that analyzed the effects of low-load RT (LL-RT) with short-term (1–3 weeks) and high weekly frequency of BFR on muscle hypertrophy and strength were not included (Ma et al., 2024). Furthermore, some studies that compared LL-RT with BFR *versus* high-load resistance training (HL-RT) were also not included (Kim et al., 2017; Galvao Pereira et al., 2019; Jessee et al., 2018; Buckner et al., 2020; Libardi et al., 2015; May et al., 2022). Given the eligibility criteria, it seems that including these studies could have provided a more comprehensive review. The absence of these studies suggests that there might be gaps in the selection process, which warrants careful interpretation of the results.

The search strategy adopted by the authors may justify the absence of certain studies. The combination of terms with the help of the Boolean operator “AND” may have limited the searches to studies that presented all the descriptors presented, including “resistance training”, hypoxia and “blood flow restriction therapy” and the respective alternative terms adopted for each descriptor. Therefore, a study that presented only the terms “resistance training” and “blood flow restriction” may not be retrieved when adopting the search strategy adopted by Ma et al. (Aromataris and Pearson, 2014).

Another point that caught our attention is the fact that the authors seem to use the terms “muscle thickness” and “cross-sectional area” (CSA) as synonyms. Muscle thickness refers to the distance between a superficial and deep border of a muscle that is usually measured at specific sites along the muscle using ultrasound imaging (Miyachi et al., 2020). On the other hand, muscle CSA refers to the total area of muscle that is perpendicular to its length (Miyachi et al., 2020). Muscle CSA is typically assessed via magnetic resonance imaging or computer tomography and is thought to present a more accurate measure of total muscle size. In essence, muscle thickness provides a 2D analysis of a measure of muscle size at a particular point in the muscle belly whereas muscle CSA provides a 3D image of the total muscle size.

### Risk of bias

The risk of bias in the studies included in the SR by Ma et al. (Ma et al., 2024) was assessed using the Cochrane Risk of Bias 2 (RoB 2) tool. The RoB two was used in a SR conducted by our research group, which compared the effect of LL-RT with BFR *versus* HL-RT (de Queiros et al., 2024).

Considering that our review had similar objectives to those of Ma et al. (Ma et al., 2024), some studies were included in both. Interestingly, there are inconsistencies between the reviews regarding the assessments of the risk of bias of these studies. In domain one of RoB 2 (bias due to the randomization process), the risk of bias rating was considered “low” in the SR of Ma et al. (Ma et al., 2024) for certain studies (Biazon et al., 2019; Centner et al., 2019; Laurentino et al., 2022; Reece et al., 2023; Ozaki et al., 2013), whereas in our review, such studies were rated as “some concerns”. In our study, this rating is justified by the fact that none of these studies detailed the randomization process or mentioned allocation concealment.

In domain 4, biases related to outcome measurement, inconsistencies were also reported; Ma et al. (Ma et al., 2024) classified all studies included in their review as “low risk of bias”. However, some studies did not report blinding of outcome assessors (Biazon et al., 2019; Laurentino et al., 2022; Reece et al., 2023; Ozaki et al., 2013; Vechin et al., 2015; Lixandrão et al., 2015). Considering the information presented, we speculate that the risk of bias assessment performed by Ma et al. (Ma et al., 2024) are not representative of the true risk of bias in the included studies.

### Meta-analyses

When analyzing the characteristics of the studies included in the SR conducted by Ma et al. (Ma et al., 2024), it is possible to identify that some studies performed comparisons between LL-RT with BFR *versus* HL-RT, while other studies compared LL-RT with BFR *versus* LL-RT without BFR. We identified that the authors included all studies in a single meta-analysis, both for strength and muscle size. This could potentially obscure the effects of resistance training with BFR and impact the generalizability of the findings.

## Discussion

The SR conducted by Ma et al. (Ma et al., 2024) might have excluded some eligible studies, which could affect the comprehensiveness of the review. In addition, we speculate that there are problems with the assessment of the risk of bias of the studies included in the review, which may lead to misleading conclusions about the quality of the evidence presented. Finally, we assert that the quantitative synthesis of the results of the studies was not done adequately, since the authors did not stratify the results according to the comparator. Therefore, we recommend that readers interpret the results with some caution, considering the potential limitations.
